# Privacy Practices of Health Information Technologies: Privacy Policy Risk Assessment Study and Proposed Guidelines

**DOI:** 10.2196/26317

**Published:** 2021-09-16

**Authors:** Haley M LaMonica, Anna E Roberts, Grace Yeeun Lee, Tracey A Davenport, Ian B Hickie

**Affiliations:** 1 Brain and Mind Centre The University of Sydney Camperdown Australia

**Keywords:** privacy, mental health, technology, digital tools, smartphone, apps

## Abstract

**Background:**

Along with the proliferation of health information technologies (HITs), there is a growing need to understand the potential privacy risks associated with using such tools. Although privacy policies are designed to inform consumers, such policies have consistently been found to be confusing and lack transparency.

**Objective:**

This study aims to present consumer preferences for accessing privacy information; develop and apply a privacy policy risk assessment tool to assess whether existing HITs meet the recommended privacy policy standards; and propose guidelines to assist health professionals and service providers with understanding the privacy risks associated with HITs, so that they can confidently promote their safe use as a part of care.

**Methods:**

In phase 1, participatory design workshops were conducted with young people who were attending a participating *headspace* center, their supportive others, and health professionals and service providers from the centers. The findings were knowledge translated to determine participant preferences for the presentation and availability of privacy information and the functionality required to support its delivery. Phase 2 included the development of the 23-item privacy policy risk assessment tool, which incorporated material from international privacy literature and standards. This tool was then used to assess the privacy policies of 34 apps and e-tools. In phase 3, privacy guidelines, which were derived from learnings from a collaborative consultation process with key stakeholders, were developed to assist health professionals and service providers with understanding the privacy risks associated with incorporating HITs as a part of clinical care.

**Results:**

When considering the use of HITs, the participatory design workshop participants indicated that they wanted privacy information to be easily accessible, transparent, and user-friendly to enable them to clearly understand what personal and health information will be collected and how these data will be shared and stored. The privacy policy review revealed consistently poor readability and transparency, which limited the utility of these documents as a source of information. Therefore, to enable informed consent, the privacy guidelines provided ensure that health professionals and consumers are fully aware of the potential for privacy risks in using HITs to support health and well-being.

**Conclusions:**

A lack of transparency in privacy policies has the potential to undermine consumers’ ability to trust that the necessary measures are in place to secure and protect the privacy of their personal and health information, thus precluding their willingness to engage with HITs. The application of the privacy guidelines will improve the confidence of health professionals and service providers in the privacy of consumer data, thus enabling them to recommend HITs to provide or support care.

## Introduction

### Health Information Technologies

Digital health has quickly become an integral component of best practice health care, transforming the way care is delivered. By capitalizing on digital infrastructure, it is widely recognized that digital health solutions improve access to care, particularly for individuals with mobility or transport restrictions, or for those who live remotely where health care resources may be limited [1,2]. The availability of health information technologies (HITs) is proving invaluable during the COVID-19 pandemic, where face-to-face mental health care is often delivered digitally (eg, videoconferencing) [3]. Beyond access issues, however, digital health also has the potential to optimize or eliminate waitlists and facilitate routine outcome monitoring to strengthen and maintain patient-health professional relationships [4], allowing for shared, data-driven decision-making on appropriate treatment plans [5]. With a greater need for and reliance on digital health solutions for screening, treatment, and ongoing maintenance of health, there is now an increased focus on the privacy and security of personal and health information collected via HITs, such as health-related apps and e-tools (eg, websites and web-based courses).

### Legal and Ethics Rights of Individuals

It is crucial to consider the legal and ethical rights of individuals who choose to both explicitly and passively share their web-based health information. This is essential, particularly in the area of mental health care, where data often contain highly personal information that could cause significant harm and distress if not handled appropriately. There is increasing documentation and guidance in this area, such as the recent release of the National Safety and Quality Digital Mental Health Standards (consultative draft) by the Australian Commission on Safety and Quality in Health Care [6], which includes educational brochures that provide tips to consumers, caregivers [7], and clinicians [8] on choosing a digital mental health service. Specific to privacy, the Australian Privacy Principles require that all organizations have a clearly expressed and current privacy policy detailing how personal information is managed [9]. As personal and health information is deemed particularly sensitive, extra protection concerning its handling was established under the Privacy Act 1988 [10]. The World Economic Forum also has highlighted *trust* as one of the primary issues that needs to be addressed on a global scale to ensure consumers’ and health professionals’ trust in the privacy and security standards of new digital tools and technology-based therapies [11].

### Privacy Practices of HITs

The use of health-related apps has rapidly increased in recent years, with 47% of Australian consumers using apps in 2018 [12]. Health professionals are also increasingly recommending HITs as part of clinical practice. For example, approximately half of Australian general practitioners responding to an annual technology survey indicated that they recommend HITs for at least monthly use by patients, with mindfulness and mental health apps recommended most often [13]. Although HITs, including apps and e-tools, have gained considerable favor with consumers and health professionals for promoting self-management of health and well-being, the privacy of personal and health information remains a notable area of concern. A systematic review of 79 health and wellness apps certified as clinically safe and trustworthy by the United Kingdom National Health Service Health Apps Library found that 66% of apps transmitting personal information on the internet did so without encryption, and 20% did not have any form of privacy policy [14]. Furthermore, although no app collected or shared information in a manner that was not explicitly stated in the privacy policy, the nature of the personal information included in such transmission was not described in 78% of policies [14]. A recent cross-sectional assessment of 36 top-ranked apps found that 92% of them transmitted data to a third party; however, only 64% of privacy policies made this explicit. In addition, only 43% and 50% of privacy policies disclosed that apps were transmitting data to Google and Facebook, respectively [15]. This begs the question, can users trust that personal and health information collected via HITs will be kept private and secure?

### Objectives

This study aims to use co-design methodologies to better understand young people’s preferences for learning about how their personal and health information will be handled by HITs and create prototypes for the InnoWell Platform. The InnoWell Platform was developed by InnoWell, a joint venture between the University of Sydney and PricewaterhouseCoopers (PwC; Australia) through Project Synergy, an Aus $30 million (US $22.1 million) Australian government–funded initiative [16]. As described previously [5,17], the InnoWell Platform is a co-designed digital tool embedded within traditional in-clinic and web-based mental health services to support person-centered, measurement-based care. This study also seeks to develop and apply a privacy policy risk assessment tool to assess whether existing HITs meet the recommended privacy policy standards and present guidelines to assist health professionals and service providers to ask the appropriate questions for themselves and HIT manufacturers to ensure that they can confidently promote the safe use of HITs as part of care.

## Methods

### Phase 1

#### Participatory Design Workshops

Participatory design (ie, co-design) methodologies are routinely used to ensure that digital tools are designed to meet the needs of the intended user base, thus increasing uptake and engagement [18,19]. Our research team has extensive experience in the use of participatory design, including workshop design and facilitation as well as knowledge translation [19-23].

Our research team conducted a series of 10 participatory design workshops from July to September 2018 in 9 urban and rural *headspace* centers across Australia (Ashfield, Bathurst, Broken Hill, Dubbo, Orange, Wagga Wagga, and Wollongong, New South Wales [NSW]; Townsville, Queensland; Edinburgh North, South Australia). *headspace* centers are primary mental health services providing support to young Australians and their families to promote mental health and engagement with the community. The methods and results of these workshops were previously reported in detail by Cheng et al [24]. In summary, the workshops brought together key stakeholders from the participating *headspace* communities, including help-seeking young people, supportive others, health professionals, and service providers, to collaboratively discuss technology designs, ideas, and principles to support mental health and well-being. In these workshops, technology designs, ideas, and principles, including the concepts of data privacy and security, were evaluated by the participants. Due to varying numbers of participants and other contextual factors, workshops ranged from 2.5 to 4 hours in duration and consisted of the following stages: discovery, evaluation, and prototype. The discovery stage focused on current ways in which technology is used by participants, including for the purposes of supporting mental health, internet access issues in regional communities, and concerns about sharing personal and health information via web-based programs or tools. During the evaluation stage, participants were presented with images of components of the InnoWell Platform and asked to document their feedback. Finally, in the prototype stage, participants were given the opportunity to brainstorm new items, functionalities, and wireframes using sketchbooks for the components or functionalities of the InnoWell Platform. In this paper, we present findings related to the development and inclusion of privacy information in HITs as co-designed by the participants. Importantly, these results were not included in the original publication [24].

#### Participants

Participants included individuals from the participating *headspace* communities, including young people attending a participating *headspace* center, a supportive other of a young person attending a participating *headspace* center (eg, family member, caregiver, or friend), or a health professional or service provider working at a participating *headspace* center. The inclusion criteria for participation in the study required participants to be aged ≥12 years, proficient in reading and speaking English, and having completed the participant consent process. Details of recruitment, screening, and informed consent processes have been previously documented by Cheng et al [24].

#### Knowledge Translation

The InnoWell Platform consists of a multidimensional assessment evaluating a range of biopsychosocial domains (eg, psychological distress, sleep, alcohol use, and physical health) to provide a holistic view of the consumer. The assessment results are available in real time and designed to be reviewed collaboratively by the consumer and their health professional to promote shared decision-making about care options, accounting for consumer preferences. A consumer’s progress can then be routinely tracked and monitored over time using assessment tools to inform treatment planning, clinical review, and coordinated care within and between services.

As previously described [24], workshop notes and descriptive artifacts were reviewed by an independent knowledge translation team with 2 young researchers without previous knowledge of the InnoWell Platform or the fundamental principles underpinning its design. Each team member, taking note of their general observations, reviewed all data independently and subsequently identified the key concepts noted by workshop participants to then produce prototype designs of the components of the InnoWell Platform.

#### Ethics

This study was approved by the University of Sydney’s Human Research Ethics Committee (protocol number: 2018/130).

### Phase 2

#### Development of the Privacy Policy Risk Assessment Tool

Drawing from digital health privacy and security criteria published by existing research and professional associations, our research team developed a privacy policy risk assessment tool (Multimedia Appendix 1) to evaluate privacy policies for HITs, including apps and e-tools. Developed in 2018 by a multidisciplinary expert panel of health professionals, informaticists, medical students, and consumers with lived experience of mental illness, the American Psychiatric Association app evaluation model includes risk, privacy and safety questions as part of their simple four-stage hierarchal app review process [25]. Although the American Psychiatric Association model served as the primary reference, to ensure an all-encompassing assessment tool, we also incorporated details from the National and Safety Quality Digital Mental Health Standards developed by the Australian Commission on Safety and Quality in Health Care to improve the quality of digital mental health care and protect the privacy of service users [6]. The final privacy policy risk assessment tool consists of 23 items covering (1) privacy policy (6 items), (2) personal health information (PHI; 4 items), (3) data security and storage (9 items), and (4) other aspects of privacy (4 items). As the questions and concepts included in the privacy policy risk assessment tool were drawn from previously published privacy and security standards, we were confident that the measure has face and construct validity; however, a specific validity analysis was not conducted.

#### Review of Digital Health Tool Privacy Policies

Importantly, the privacy policy risk assessment tool is broadly applicable to HITs. To demonstrate its utility, in this study, we evaluated the privacy policies of the apps and e-tools in the *youth* configuration of the InnoWell Platform [16]. Within the InnoWell Platform, there are two types of care options: clinical and nonclinical. Clinical care options require health professionals’ involvement, such as individual therapy and group therapy, whereas a consumer can immediately access and begin using nonclinical care options, such as apps and e-tools, without the support of a health professional [26]. During the co-design process, care options are tailored to the consumer population; in this case, young people receiving care through primary youth mental health services (eg, *headspace* centers).

Aligned with established evaluation processes [15], privacy policies and any related material that may contain privacy-related content, such as terms and conditions, were sourced from associated websites and app store links. A nonrestrictive process was used, allowing all hyperlinks from the app store or within the privacy policy to be considered. All available information was collated and reviewed in accordance with the privacy policy risk assessment tool developed primarily by a member of the research team (AER), with support from a research intern (Toby Wong).

#### Assessing Readability

The readability levels of each privacy policy were assessed as part of the evaluation process. There are multiple readability formulas available; however, for the purpose of this study, we used the Flesh-Kincaid readability tests because of their acceptance in the health care literature [27]. The readability tests were designed to indicate how difficult an English passage of writing is to understand using a formula calculated from the average number of syllables per word and the average number of words per sentence [28]. Both the Flesch reading ease and the Flesch-Kincaid reading grade level were calculated using the web-based readability tools [29]. The scores indicate the readability of a passage of text on a scale from “very easy to read" to "very difficult to read” and “fifth grade reading level” to “professional reading level,” respectively. Scores were calculated using the first two paragraphs of the privacy policies. The paragraphs (at least 100 words) were copied into a readability formula calculator, and a score was given.

#### Data Analysis

Descriptive statistics were used to analyze all aspects of the assessment data. SPSS version 25 (IBM Corp) was used for all analyses.

### Phase 3

#### Development of Privacy Guidelines: Consultations

As a result of Project Synergy and the development of the InnoWell Platform, a set of core principles and privacy guidelines were used as the starting point to formalize a more encompassing set of privacy guidelines. A series of consultations were held between 2014 and 2016 (phase 1) and then again between 2017 and 2018 (phase 2) to develop a set of privacy guidelines for Project Synergy. Initial consultations were conducted by Orygen (the National Centre of Excellence in Youth Mental Health) [30]. The subsequent consultations were conducted by the Project Synergy research and development team (led by the authors TAD and IBH). These consultations were held at the University of Sydney’s Brain and Mind Centre, either in-person or via teleconference, and brought together key stakeholders across relevant organizations.

#### Participants

Participants included key stakeholder groups, including Orygen (the National Centre of Excellence in Youth Mental Health), the Young and Well Cooperative Research Centre, Mental Health Commission of NSW (Pacific Privacy Consulting Pty Ltd), the Project Synergy research and development team (the University of Sydney’s Brain and Mind Centre), InnoWell, and PwC (Australia). Select individuals were nominated by each organization, with participants contributing diverse expertise and experience, such as the involvement of 2 ex-serving privacy commissioners for NSW and Victoria (Australia).

#### Preliminary Development of Privacy Guidelines for Phase 1 Project Synergy (2014-2016)

From the outset of the development cycle of the prototype and as part of phase 1 of Project Synergy (2014-2016) [16], this ongoing series of consultations were envisaged to inform the development of the privacy guidelines. Therefore, an initial set of guidelines was developed; these guidelines were produced by Orygen and supported by the Young and Well Cooperative Research Centre and the Mental Health Commission of NSW [30]. They were also reviewed by the Project Synergy research and development team at the University of Sydney’s Brain and Mind Centre (the authors) and Pacific Privacy Consulting.

#### Ongoing Development of Privacy Guidelines for Phase 2 of Project Synergy (2017-2020)

As a result of the review of the initial guidelines developed in phase 1 of Project Synergy, a narrower focus was decided upon and used as the starting point for the development of more encompassing privacy guidelines for phase 2 of Project Synergy (2017-20). Specifically, upon review of the initial guidelines whereby the University of Sydney and Pacific Privacy Consulting determined that privacy concerns were the most important priority, a narrower focus was given to 8 core foundation principles to be followed by organizations using the prototype in phase 1 of Project Synergy (2014-2016) [16]. The core foundation principles included responsibility for legal compliance, anonymous or pseudonymous services wherever practicable, individual control, transparency, interaction with individuals, encryption, deidentification, and cross-border processing risk. Development of the broad structure and content of the guidelines was guided by the Project Synergy research and development team, with review and input by Pacific Privacy Consulting and InnoWell.

This paper presents privacy guidelines to assist health service providers in considering the privacy of their consumers when using HITs as part of care. The guidelines were first drafted by the Project Synergy research and development team based on the information gathered through the initial collaborative consultation process. The checklist was then reviewed, discussed, and evaluated by the research team, ultimately resulting in agreement by consensus.

## Results

### Knowledge Translation Findings

The results of the knowledge translation process highlighted that participants wanted privacy information to be presented before being required to create an account. Specifically, they emphasized the need for privacy information to be readily available, allowing a user to be completely comfortable when entering more sensitive information into a HIT, such as the InnoWell Platform (eg, “Always ask could this site be more secure with my information” [Wollongong workshop]). This included the ability to change permissions concerning data sharing at their discretion (eg, “[I] would want privacy settings in place so that not everyone that shares the system can see” [Broken Hill workshop]). Participants noted that privacy information is frequently confusing and difficult to understand, leaving them unsure whether they should trust the HIT to protect their personal and health information. Thus, multiple participants suggested a pin code or password (eg, “Consider password security like in bank apps.” [Townsville workshop]) to access certain data so the consumer controls who has access to their information in the InnoWell Platform. Importantly, the idea of consumer control extended beyond HIT manufacturers such as InnoWell and included health professionals and supportive others (ie, family members and carers) accessing personal and health information (eg, “Need privacy setting like Facebook...can filter who can see the information” [Bathurst workshop]).

### App and e-Tool Privacy Policy Assessment

We evaluated 34 privacy policies using the privacy policy risk assessment tool. Most of these apps and e-tools were designed for both youth and adult users (28/34, 82%), whereas the remaining 18% (6/34) were specifically designed for youth audiences (aged ≤25 years). Most apps and e-tools (20/34, 59%) were self-help or self-management tools supporting mental health and well-being, including three specifically using cognitive behavioral therapy techniques. There were also 12% (4/34) symptom trackers, 6% (2/34) web-based counseling services, 6% (2/34) planning and time management tools, 6% (2/34) psychoeducational websites, and 2% (1/34) mindfulness and meditation app. The remaining apps and e-tools supported fitness (2/34, 6%) and relationships (1/34, 2%).

### Summary of Privacy Policy Information

#### Overview

The summary results from the review of privacy policies are presented in Table 1. All apps and e-tools had privacy policies. Some of the policies were not readily accessible directly from the app or e-tool but rather were hosted on an external website that the app or e-tool privacy policy fell under (ie, a privacy policy for a hospital or government department). Importantly, most of the policies (26/34, 76%) explicitly stated that they met the standards of the Privacy Act 1988 (Australia) or international equivalent (ie, Health Insurance Portability and Accountability Act). In addition, most manufacturers (31/34, 91%) introduced the purpose of the privacy policy, stating that the policy explained the manufacturers’ approach to privacy, protection, and management of personal information. Similarly, 97% (33/34) of privacy policies were noted to provide adequate information for all potential users. In contrast, manufacturers (26/34, 76%) frequently did not provide adequate information about their organization and how or why they operated.

**Table 1:** Summary results of the privacy policy assessment results (N=34).

Privacy policy questions and responses			Value, n (%)
**Is there a privacy policy?**			
	No	0 (0)	
	Yes	34 (100)	
**Does the app or e-tool claim to meet the standards of the Privacy Act 1988 (Australia), HIPAA^a^** **(United States), or another international equivalent?**			
	No	8 (24)	
	Yes	26 (76)	
**Did the manufacturer introduce the purpose of the privacy policy?**			
	No	3 (9)	
	Yes	31 (91)	
**Does the privacy policy provide an introduction to the organization, including its vision and purpose?**			
	No	26 (76)	
	Some information	3 (9)	
	Yes	5 (15)	
**Does the privacy policy provide adequate information (both targeted and general) relevant for all users, including consumers seeking care and health professionals?**			
	No	0 (0)	
	Some information	1 (3)	
	Yes	33 (97)	

^a^HIPAA: Health Insurance Portability and Accountability Act.

#### Readability of Privacy Policies

In relation to the Flesch reading ease, the privacy policies were all found to fall into the top three of the seven available score categories, with 9% (3/34) rated as *fairly difficult to read*, an additional 53% (18/34) rated as *difficult to read*, and the remaining 38% (13/34) rated as *very difficult to read.* The Flesh-Kincaid grade-level test illustrated similar results with only the top two levels represented out of eight possible levels. All but three policies fell into the *college graduate* grade level (31/34, 91%), with the remaining considered *professional-level* reading difficulty (3/34, 9%).

#### Collection of PHI

Table 2 presents results from the PHI assessment. Most apps and e-tools (32/34, 94%) collected some form of PHI, ranging from simple demographic information to more sensitive information such as information related to mental health. The remaining 6% (2/34) of apps and e-tools were considered informational tools and collected data such as email addresses. Of the 32 apps and e-tools that collected PHI, most (28/32, 87%) shared this information in some manner (data sharing is reviewed in greater detail below). This use was disclosed in most privacy policies (27/32, 85%), explicitly stating that reasonable steps were taken to ensure the security of the PHI; however, for 6% (2/32) and 9% (3/32) of privacy policies how PHI was shared was *somewhat clear* or *not clear*, respectively. Although more than half of the privacy policies (17/32, 53%) clearly stated how and when PHI was deleted, this information was either *somewhat clear* or *not clear* in the remaining 3% (1/32) or 44% (14/32) privacy policies, respectively.

**Table 2:** PHI^a^ assessment results (N=34).

PHI questions and responses			Value, n (%)
**Does the app or e-tool collect PHI (ie, demographic information, medical histories, test and laboratory results, mental health conditions, or insurance information)?**			
	No	2 (6)	
	Yes	32 (94)	
**Is PHI (ie, demographic information, medical histories, test and laboratory results, mental health conditions, or insurance information) shared?**			
	No	4 (13)	
	Yes	28 (87)	
**Is it clear if the organization has taken reasonable steps to ensure the security of PHI?**			
	Not clear	3 (9)	
	Somewhat clear	2 (6)	
	Yes**—**clear	27 (85)	
**Is it clear how and when their organization will delete PHI?**			
	No	14 (44)	
	Somewhat clear	1 (3)	
	Yes	17 (53)	

^a^PHI: personal health information.

#### Data Sharing and Use of Information

Results of the assessment questions related to data sharing, data preferences, and data storage are presented in Table 3. Most privacy policies (32/34, 94%) declared how data were used and for what purposes, with approximately two-thirds (22/34, 65%) stating that users were allowed to delete data. Notably, only 3% (1/34) of apps allowed data sharing preferences to be changed, although this required the user to contact the manufacturer via email; 9% (3/34) of apps and e-tools enabled users with some permissions specific to data sharing, such as receiving push notifications or allowing the user to choose whether to share data such as location with the mobile app.

**Table 3:** Data sharing and data use assessment results (N=34).

Data sharing and data use questions and responses			Value, n (%)
**Does the privacy policy declare data use and purpose?**			
	No	2 (6)	
	Yes	32 (94)	
**Is shared data deidentified? (ie, is data anonymous—is personal information masked or severed from the identity of the contributor)**			
	No	11 (33)	
	Yes	23 (67)	
**Can the user change their preferences regarding data sharing (ie, switch it on or off)?**			
	No	30 (88)	
	Yes	1 (3)	
	Some permissions	3 (9)	
**Can the user delete their data from the app or e-tool?**			
	No	10 (29)	
	Yes	22 (65)	
	Informational webpage only	2 (6)	
**Is user data stored on the device?**			
	No	27 (79)	
	Yes	4 (12)	
	Unspecified	3 (9)	
**Is user data stored on a server?**			
	Yes	32 (94)	
	Unspecified	2 (6)	
**For how long is user data stored?**			
	Unspecified or unclear	12 (35)	
	Until no longer needed	12 (35)	
	1 year or less	2 (6)	
	1-3 years	3 (9)	
	More than 3 years	3 (9)	
	At user discretion	2 (6)	
**What type of server is used to store user data? (eg, Amazon Web Services, within Australian borders)**			
	Secure Australian server	6 (19)	
	Secure overseas server	11 (34)	
	Unspecified server	7 (22)	
	University server	2 (6)	
	Hospital or PHN^a^	2 (6)	
	Unclear	4 (13)	
**In what country is the server located** **?**			
	Australia	13 (38)	
	United States	9 (26)	
	Canada	3 (9)	
	Europe	1 (3)	
	Multiple countries	2 (6)	
	Unclear or unspecified	6 (18)	

^a^PHN: Primary Health Network.

Most apps and e-tools (32/34, 94%) stored data on a server, with a small number (4/34, 12%) storing data on both the device (ie, PHI) and a server (ie, email address and website activity). In addition, 6% (2/34) of apps and e-tools did not specify where the data were stored. Data storage duration ranged from up to 1 year (2/34, 6%), 1 to 3 years (3/34, 9%), and more than 3 years (3/34, 9%). Although more than one-third of the apps and e-tools were unclear or did not specify for how long data were stored (12/34, 35%), approximately one-third (12/34, 35%) stored the data until no longer needed by the organization. The remaining 6% (2/34) of privacy policies stated that the data would be deleted at the user’s discretion.

Of the 32 apps and e-tools that stored data on a server, 11 (34%) stored data on a secure overseas server, 6 (19%) stored data on a secure Australian server, 7 (22%) stored data on an unnamed or unspecified server. In addition, 6% (2/32) of apps and e-tools stored data on a university server, 6% (2/32) stored data on a hospital or primary health network server system, and 13% (4/32) were unclear on the type of server used. The location of data storage was mixed between Australia (13/34, 38%), the United States (9/34, 26%), Canada (3/34, 9%), Europe (1/34, 3%), and multiple locations (2/34, 6%), with the remainder unclear as to where data were stored (6/34, 18%).

#### Review of How Data Are Shared

Most apps and e-tools (27/34, 79%) shared data with relevant third parties, including but not limited to partners, suppliers, collaborators, advisers, and business associates. The types of data shared varied from PHI to aggregated user data, such as location. A small number of apps and e-tools (4/34, 12%) shared information with irrelevant third parties, including social media platforms such as Facebook. In addition, 38% (13/34) of apps and e-tools shared data with a research partner or university, 15% (5/34) shared information with government departments, and 38% (13/34) shared data with a health-related group or person (eg, a support person or health professional).

#### Additional Information

All privacy policies were assessed for their inclusion of various other details, which are summarized in Table 4. More than half of the apps and e-tools (20/34, 59%) used third-party vendors, such as Google Analytics, to evaluate and track consumer use of websites, collect demographic data, and evaluate other information related to the apps or e-tool website and the user’s device. Less than one-quarter of privacy policies (8/34, 24%) adequately explained how the manufacturer would respond to a data breach, although most policies (31/34, 91%) provided some details as to how to provide feedback or lodge a complaint either with the manufacturer or through an expert third party (ie, Office of the Australian Information Commissioner). Finally, only 24% (8/34) of privacy policies explicitly warned individuals about privacy risks involved in accessing services that are outside the control of the service provider, such as third-party advertisements, with an additional 9% (3/34) of policies providing some detail in this regard.

**Table 4:** Overview of additional details provided by privacy policies (N=34).

Additional questions and responses			Value, n (%)
**Does the app or e-tool use third-party vendors (eg, Google Analytics)?**			
	No	11 (32)	
	Yes	20 (59)	
	Unspecified	3 (9)	
**Is the manner in which the organization will respond to a data breach adequately explained?**			
	No	26 (76)	
	Yes	8 (24)	
**Does the privacy policy inform users how they can make inquiries and provide feedback and lodge complaints, including both contact details for the relevant party within the organization and a third-party expert (eg, Office of the Australian Information Commissioner)?**			
	No—does not provide either	3 (9)	
	Some—provides information for internal or third party only	16 (47)	
	Yes—provides both internal and third-party expert	15 (44)	
**Does the privacy policy explicitly warn users about privacy risks involved in accessing services offered that are outside the control of the organization** **?**			
	No	23 (67)	
	Somewhat	3 (9)	
	Yes	8 (24)	

### Privacy Considerations for Health Services

As generated through the collaborative consultation process described previously, Textbox 1 presents privacy guidelines to assist health professionals and service providers to ask the appropriate questions—of themselves and to HIT manufacturers—before confidently promoting the safe use of HITs as part of mental health care.

**Privacy guidelines—health professional and service provider considerations regarding the use of health information technologies (HITs) for care.**

**Privacy guidelines**

- The HIT manufacturer has clearly introduced the purpose of its privacy policy.
- The privacy policy includes an introduction to the HIT manufacturer and how and why their organization operates.
- The privacy policy provides adequate information and addresses my concerns.
  If no, I am aware who I need to contact to seek clarification...
- The privacy policy is written clearly.
  If no, I am aware who I need to contact to seek clarification...
- The privacy policy adequately explains how the HIT manufacturer will collect and use personal data.
- The privacy policy adequately explains how and when the HIT manufacturer will disclose personal data to third parties.
  If the HIT manufacturer shares data with third parties, I am confident that the third-party partners are reputable and will comply with all legislative requirements when collecting, storing, and sharing data.
  If no, I am aware who I need to contact to seek clarification...
- I am confident the HIT manufacturer has taken the appropriate steps to protect everyone’s data, adopting the strongest security measures.
- I have been made aware of how end users can access and correct their personal information on the HIT.
  If no, I am aware who I need to contact to seek clarification...
- The privacy policy outlines how and when the HIT manufacturer will delete personal data.
- The privacy policy outlines how the HIT manufacturer will respond to any data breaches.
- The privacy policy includes information on how I can inquire, provide feedback, or make complaints.
- There is the opportunity for me to contact a third-party expert to inquire about the privacy policy (such as the Office of the Australian Information Commissioner).
  If yes, they are...
- From what I read, I feel comfortable using the proposed HIT as part of my clinical care and/or practice.

## Discussion

### Listening to Consumers

With their increased experience and exposure, consumers are becoming more sophisticated users of HITs. They can offer valuable insights into how privacy information should be presented to ensure that it is clear, informative, and transparent. Participants of our co-design workshops highlighted the need for all HITs to have a privacy policy that provides relevant data security information before collecting information from an individual, preceding the account creation process. In addition, participants stated that privacy policies should be accessible, transparent, and user-friendly, ensuring that consumers understand what personal and health data will be collected, stored, and shared and, in turn, enabling them to trust the HIT to protect their data. These findings align with those reported by Schueller et al [31], which indicated that 70% of mental health app users rate the inclusion of a privacy policy and data encryption as important. Of note, when a mental health app was deemed to be from a *trusted source* (no shared definition of what constituted a trusted source), users assumed that the app adequately protected their data [31], potentially leaving their data vulnerable to unrecognized data sharing pathways.

### Privacy Policy Risk Assessment

As evidenced by our co-design results, consumers are calling for greater clarity and transparency in the privacy policies of HITs so that they can be confident that they understand how their personal and health information may be used. Importantly, all apps and e-tools included in this study had a privacy policy. All but 1 of those policies provided explicit details explaining the manufacturer’s approach to privacy and how personal information is managed and protected. However, approximately one-quarter of the privacy policies did not meet the standards of the Australian Privacy Act 1988 or other international equivalents, raising concerns regarding undisclosed data sharing and poorly secured data storage. Even when the use of data adheres to privacy standards, issues of transparency often arise. For example, a recent review of the data sharing practices of 24 health-related apps found that data were shared with 55 unique entities, including app developers, their parent companies, and third parties (ie, service providers). Subsequently, third parties shared user data with an additional 216 integrated fourth parties (eg, Facebook sharing data with data brokers to enable targeted advertising) [32].

In addition to poor overall transparency, our results also confirm that privacy policies, when present, are fairly difficult to read and require a college or professional reading level, essentially rendering them useless for a large portion of potential users (eg, children and young people or individuals with cognitive impairments or intellectual disabilities). This aligns with previous research by Robillard et al [33], which highlighted that the readability of privacy policies of mental health apps is typically written at too high a level for the general population. Strikingly, a longitudinal review of privacy policies found both a decline in readability and a marked increase in length [34]. To improve the readability of privacy policies, it is suggested that HIT manufacturers either compare different versions of their policy to determine which one has the best readability score or that the policy be rewritten until it meets a predetermined grade level. Researchers have suggested that a grade 9-10 reading level is likely to be appropriate for the general population [35].

Most apps and e-tools included in this study collected some form of PHI (32/34, 94%), including, in some cases, information related to mental health, with 87% (28/34) of those apps and e-tools then sharing these data in some manner. Although data sharing was disclosed in most privacy policies (27/32, 84%), how PHI was shared was not transparent in 15% (5/32) of the policies. Most apps and e-tools shared data with relevant third parties (27/34, 79%); however, 12% (4/34) also shared information with third parties deemed to be irrelevant, such as social media platforms (ie, Facebook). Of note, few apps and e-tools (4/31, 13%) allowed users to update their permissions concerning data sharing.

Although it is unlawful in Australia, for example, to share PHI for purposes other than those stated in a privacy policy, the complexities of the web-based environment frequently preclude a full understanding of how data are shared and for what purpose [36]. Users must recognize that shared data enters into a *supply chain*, being passed from apps and e-tools to parent companies and then on to third parties such as data trackers, aggregators, and brokers [37]. Subsequently, data may be sold to researchers and government agencies for advertising purposes. In addition to driving targeted advertising campaigns, this aggregated data may also be used to influence employment and financial and insurance decisions, with potentially marked consequences at the individual level [37], potentially leading to incarceration or human rights abuses in some countries [11]. Given the risks described above, explicit and transparent documentation of how data are shared is critical to ensure that users are able to provide informed consent. Furthermore, it is critical that governance structures and regulatory standards are established globally to ensure ethical practice in digital mental health care, including the handling of PHI. As advised by the World Economic Forum, regulations should not be designed exclusively by governments but rather in collaboration with consumers with lived experience, mental health professionals, technology manufacturers, and policy makers, with the aim of facilitating efficient access to effective and safe digital tools to address growing mental health needs [11].

Most apps and e-tools in our sample stored data on a server (32/34, 94%), with more than half (21/32, 66%) storing data on an unnamed or unspecified server. Although other server types included university servers (2/32, 6%) and a hospital or primary health network server system (2/32, 6%), the type of server used to store data for an additional 22% (7/32) of apps and e-tools was unclear. Once data are transmitted to a third-party server, it is often difficult to determine the robustness of the privacy and confidentiality standards in place to protect the PHI. For example, Cultura Colectiva, a digital media company with access to user information from Facebook, stored data on a publicly accessible server, resulting in the exposure of 540 million individual records, including user IDs and names [38]. In addition, although it is ideal that all information being transmitted is encrypted, personal and health information may still be visible in server logs with few restrictions in terms of access [39]. Notably, when different apps and e-tools use the same server, it may be possible to link different PHIs together to create digital profiles of users [40] with potentially negative repercussions such as impacts on employment and insurance. Despite the frequency of data breaches [41], 76% (26/34) of the privacy policies reviewed in this study failed to document how the organization would respond to data breaches, leaving the user to wonder what steps might be taken to protect their data from exposure and misuse (ie, identify theft).

### Privacy Guidelines for Health Professionals and Service Providers

Given the concerns regarding accessibility, transparency, and readability outlined above, through a consultative process with key stakeholders, our team developed privacy guidelines (Textbox 1) to prompt health professionals and service providers to ask informed questions when reviewing an HIT privacy policy to ensure adequate data privacy and security measures are in place. The guidelines aim to support health professionals and service providers to confidently promote the safe use of HITs as part of care and within the broader service. The privacy guidelines have considered the privacy policy of HIT manufacturers to emphasize the importance of building trust between users and HIT manufacturers through transparency [42]. Thus, it is the responsibility of the HIT manufacturer to be aware of all (both current and emerging) regulatory requirements and best practice principles [42] to ensure that the privacy policy is communicated to all users. This not only minimizes potential harm but also allows users to be well informed and to have more control when consenting to use HITs.

As few consumers will review academic literature before accessing HITs, they are more likely to learn about available apps and e-tools via the internet, app stores, social media, word of mouth, or health professionals. In relation to the latter, it is recommended that health professionals and service providers conduct their own risk assessment before implementing HITs into their service to ensure appropriate risk strategies are in place [3]. Not only should providers have an understanding of privacy risks but it is also important that they work with consumers to ensure they are aware of the potential for privacy breaches to ensure they are providing informed consent before engaging with an HIT [37].

### Limitations

This study has some limitations that are important to note. Although we engaged in a thorough collaborative consultation process to develop the broad structure and content of the privacy guidelines for Project Synergy, the development of the privacy policy risk assessment tool and the guidelines for health professionals and service providers was conducted by the research team, independent of this broader stakeholder group. Therefore, we acknowledge that both the assessment tool and the guidelines may benefit from further input or revision by individuals with expertise in data privacy and security, both from a legal perspective and regarding manufacturers of digital tools. As highlighted by the co-design work presented in phase 1, our group recognizes the importance of including the voice of those with lived experience in our work to reform mental health services and systems of care, including the ethical development and application of digital tools. With that being said, we acknowledge that the privacy policy risk assessment tool and guidelines were developed without contributions from consumers with lived experience of mental ill health.

### Conclusions

Given the increasing uptake of HITs, both by individuals for the purposes of self-management and by health professionals as a means to complement clinical services, it is essential that all users have a clear understanding of what personal and health information will be collected, how these data will be shared and stored, and what privacy and security measures are in place to ensure it is protected. Our findings highlight the ubiquitous poor readability and lack of transparency in existing privacy policies, a stark contrast to what consumers emphasized as essential factors in the presentation of privacy information. Although consumers, health professionals, and services are becoming increasingly reliant on HITs to deliver, support, or enhance care, concerns regarding the privacy of health and personal information are likely to undermine user confidence and willingness to engage with HITs. Therefore, we provide suggested guidelines that can be easily adopted by health professionals and service providers when considering the implementation of HITs, including apps and e-tools, into their service. We recommend that these guidelines be adopted to ensure that HITs are used to their full potential to maximize patient health outcomes while minimizing risk and that users are informed of privacy and security considerations to make educated decisions as to whether they would like to share their personal and health information.

## Appendix

Multimedia Appendix 1 Privacy risk assessment tool.

## Abbreviations

HIT: health information technology
NSW: New South Wales
PHI: personal health information
PwC: PricewaterhouseCoopers
