# Supplementary material for: Privacy Practices of Health Information Technologies: Privacy Policy Risk Assessment Study and Proposed Guidelines
Source: J Med Internet Res. 2021 Sep 16;23(9):e26317. doi: 10.2196/26317 (PMC8485195; doi:10.2196/26317)
Supplement: Multimedia Appendix 1 [file jmir_v23i9e26317_app1.docx]

## Multimedia appendix 1

Privacy Policy Risk Assessment Tool

**Privacy and Security**: Data privacy and security level that the mobile application or online tool provides.

**Privacy Policy questions:**

1. Who is the target audience (age range or population) for the app or e-tool?

   FREE RESPONSE
2. Is there a privacy policy?

   NO
   YES

**If Yes Q3; If No Q8**

1. What reading level is the privacy policy?
   (Switch on readability scores in Microsoft word: Options> Proofing > Show readability statistics. Please copy and paste the first paragraph from the privacy policy into a word document and note the Flesch reading ease and Flesch-kincaid grade level)

   Flesch reading ease:
   Flesch-kincaid grade level:
2. Does the app or e-tool claim to meet the standards of the Privacy Act 1988 (Australia), HIPAA (USA) or other international equivalent?

   NO
   YES
3. Did the manufacturer introduce the purpose of the privacy policy?

NO
YES

1. Does the privacy policy provide an introduction to the organisation, including their vision and purpose?

   NO
   SOME INFORMATION
   YES
2. Does the privacy policy provide adequate information (both targeted and general) relevant for all users, including consumers seeking care and health professionals?

   NO
   YES

**Personal health information questions:**

1. Does the app or e-tool collect personal health information (i.e. demographic information, medical histories, test and laboratory results, mental health conditions, insurance information)?

   NO
   YES

   **If Yes Q9; If No Q12**
2. Is personal health information (i.e. demographic information, medical histories, test and laboratory results, mental health conditions, insurance information) shared?

   NO
   YES
3. Is it clear if the organisation has taken reasonable steps to ensure the security of personal health information?

   NOT CLEAR
   SOMEWHAT CLEAR
   YES - VERY CLEAR
4. Is it clear how and when the organisation will delete personal health information?

   NOT CLEAR
   SOMEWHAT CLEAR
   YES - VERY CLEAR

**Data security and storage questions:**

1. Does the privacy policy declare data use and purpose?

   NO
   YES
2. With whom is the data shared (e.g. a research group or University, an external corporation or third party)?

   A. Relevant third parties (e.g. for analytic purposes, customer support, partners, suppliers, collaborators, advisers and business associates)
   B. Irrelevant third parties (e.g. Social media)
   C. Academic research partners or University
   D. Government departments
   E. Health related groups/people: service providers, health professionals, support people)
   F. Other
3. Is shared data de-identified? (i.e. is data anonymous – is personal information masked or severed from identity of the contributor)
   NO
   YES
4. Can the user change their preferences regarding data sharing (i.e. switch it on/off)?
   NO
   YES
5. Can the user delete data from the app or e-tool?
   NO
   YES
6. Is user data stored on the device?
   NO
   YES
7. Is user data stored on a server?
   NO
   YES

**If Yes Q19; If No Q21**

1. For how long is data stored?

UNSPECIFIED
UNTIL NO LONGER NEEDED
LESS THAN 1 year
1-3 YEARS
> 3 YEARS
AT USER DISCRETION

1. What type of server is used to store user data? (e.g. Amazon Web Services, within Australian borders, etc.)

   SECURE AUSTRALIAN SERVER
   SECURE OVERSEAS SERVER
   UNSPECIFIED SERVER
   UNIVERSITY SERVER
   HOSPITAL/PHN SERVER
   UNCLEAR
2. In what country is the server located?

   Australia
   America
   Canada
   Multiple Countries
   Europe
   Unclear

**Other aspects of privacy:**

1. Does the app use third party vendors (i.e. Google Analytics, etc)?

   NO
   YES
2. Is the manner in which the organisation will respond to a data breach adequately explained??

   NO
   YES
3. Does the privacy policy inform users as to how they can make enquires, provide feedback and/or lodge complaints, including both contact details for the relevant party within the organisation as well as a third-party expert (e.g. Office of the Australian Information Commissioner)?

   NO – does not provide either
   SOME - Provides information for internal or third party Only
   YES – provides both internal and third party expert
4. Does the privacy policy explicitly warn users about privacy risks involved in accessing services offered that are outside the control of the organisation?

   NO
   YES
